# One-Stage Extension Shortening Osteotomy for Syndromic Camptodactyly

**DOI:** 10.3390/jcm9113731

**Published:** 2020-11-20

**Authors:** Byoung Kyu Park, Hyun Woo Kim, Hoon Park, Min Jung Park, Kee-Bum Hong, Kun Bo Park

**Affiliations:** 1 Graduate School, Yonsei University College of Medicine, Seoul 03722, Korea; pedpbk@paik.ac.kr; 2 Department of Orthopaedic Surgery, Inje University Haeundae Paik Hospital, Busan 48108, Korea; 3 Division of Pediatric Orthopaedic Surgery, Severance Children’s Hospital, Yonsei University College of Medicine, Seoul 03722, Korea; pedhkim@yuhs.ac (H.W.K.); icefroast@yuhs.ac (K.-B.H.); 4 Department of Orthopaedic Surgery, Gangnam Severance Hospital, Yonsei University College of Medicine, Seoul 06273, Korea; hoondeng@yuhs.ac; 5 Department of Orthopedic Surgery, National Health Insurance Service Ilsan Hospital, Goyang 10444, Korea; minjung815@gmail.com

**Keywords:** camptodactyly, finger, osteotomy, children

## Abstract

Syndromic camptodactyly often affects multiple fingers, and severe deformities are common compared to idiopathic camptodactyly. This study aimed to evaluate the use of a one-stage extension shortening osteotomy of the proximal phalanx for patients with syndromic camptodactyly without tendon surgery. Forty-nine cases of syndromic camptodactyly were included. Forty fingers (81.6%) were associated with arthrogryposis multiplex congenita, and nine (18.4%) with other syndromes. Six fingers presented with a moderate form (30° to 60°) of camptodactyly, whereas 43 fingers manifested the severe form (>60°). The mean age at the time of surgery was 8.5 years, and the patients were followed for a mean of 3.9 years. The mean length of the shortening of the proximal phalanx was 4.9 mm, which averaged 17.8% of the proximal phalanx’s original preoperative length. The mean operative time was 25.8 min, and the PIP joint was fixed using Kirschner wires with an average flexion position of 7.6°. The mean flexion contracture improved from 76° preoperatively to 41° postoperatively. The mean preoperative active arc of motion was 23°, which improved to 49° postoperatively. A one-stage extension shortening osteotomy is a straightforward and effective technique for the improvement of finger function through the indirect lengthening of volar structures without the flexor tendon lengthening. The osteotomy could simultaneously correct bony abnormalities. This simple procedure is especially suitable for surgery on multiple fingers in patients with syndromic camptodactyly.

## 1. Introduction

**Figure 1:**
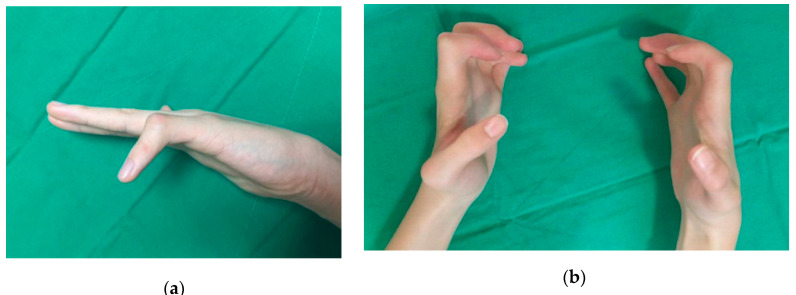
Clinical images of (**a**) a patient with idiopathic camptodactyly of a lesser digit and (**b**) a patient with syndromic camptodactyly involving multiple fingers.

Camptodactyly is the nontraumatic and progressive flexion contracture of the proximal interphalangeal (PIP) joint, which is relatively rare and occurs in less than 1% of the population [1]. The treatment of camptodactyly aims to improve functional and cosmetic outcomes. Due to all structures at the base of the finger can be involved in the pathogenesis of the deformity [2], the surgical treatment for this particular type of deformity is controversial and challenging [3,4,5,6,7]. To optimize treatment, the severity and type of camptodactyly should be considered. Benson et al. [8] divided camptodactyly into three types: infantile, adolescent, and syndromic. In idiopathic infantile and adolescent camptodactyly, the little finger is most often affected, and cosmetic complaints are more common than functional impairments (Figure 1a) [3,9,10]. However, syndromic camptodactyly often affects multiple fingers, and bilateral deformities with severe fixed contractures are common (Figure 1b) [8,11]. Syndromic camptodactyly causes more considerable discomfort and cosmetic problems than idiopathic camptodactyly. Thus, in most cases, surgery is necessary to treat this condition.

One-stage or staged soft tissue surgery is recommended for the correction of camptodactyly [3,4,5,6,7,12]. However, previous studies have shown inconsistent outcomes in terms of the degree of correction, the rate of complications, and the likelihood of recurrence. Evans et al. [7] reported a more significant extension of the PIP joint after volar soft tissue release and flexor digitorum superficialis (FDS) tenotomy, but the arc of motion did not improve over a mean follow-up period of 1.4 years. Hamilton et al. [6] suggested that a stepwise surgical approach could achieve a nearly complete resolution. However, syndromic camptodactyly was not included in their study, and the mean follow-up period of 11 months was insufficient to guarantee long-term results. Most previous studies have been limited to idiopathic camptodactyly without considering syndromic camptodactyly. Previous studies focused on soft tissue surgery to correct the contracture and did not mention bone correction. Bony changes in the PIP joint resulting from long-term contracture may not be resolved by soft tissue surgery [10,13].

Extension shortening osteotomy can be performed to lengthen the extensors and flexors and compensate for the flexion contracture [14,15,16]. Based on the treatment of arthrogryposis multiplex congenita and cerebral palsy in multiple joints, soft tissue surgeries are not sufficient to correct fixed contractures; thus, bony procedures are often required. We designed a one-stage extension shortening osteotomy to release all PIP joint structures and to reduce both damages to the volar soft tissues and weakness of the flexor tendon. Our technique differs from previously reported methods in that it does not require a volar soft tissue approach. This study aimed to introduce the index surgery for patients with syndromic camptodactyly who had difficulty grasping and a poor response to stretching. The results of the correction of the flexion contracture and the active arc of motion of the PIP joint are described below.

## 2. Experimental Section

**Figure 2:**
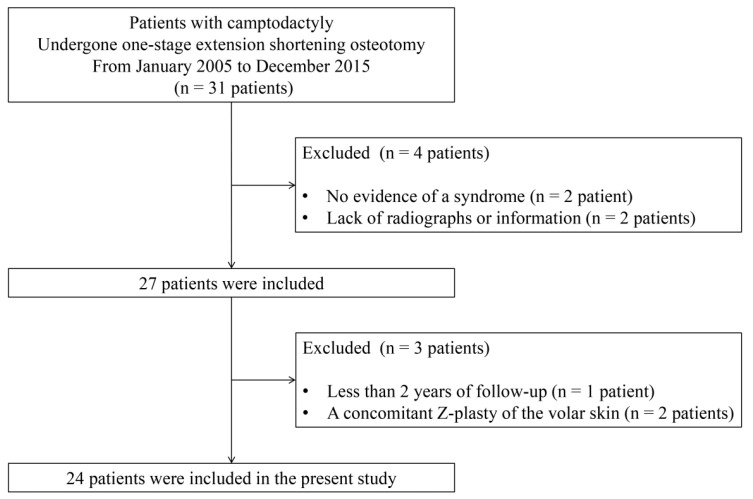
The flow diagram of the inclusion/exclusion process.

The Institutional Review Board of our hospital approved this retrospective study (IRB No. 4-2019-1236). We reviewed the medical records and radiographs of 31 patients that were camptodactyly treated using the index surgery in our tertiary children’s hospital between 2005 and 2015. All operation was performed by a senior author (H.W.K.) with expertise level 4 according to the criteria of Tang and Giddins [17]. The indication of surgery for this period was functional impairments due to flexion deformities of more than 30° that interfere with daily life in patients who had shown an inadequate response to conservative treatments, including a comprehensive multidisciplinary rehabilitation and stretching exercises. Any preoperative splinting therapy was not performed because its efficacy is uncertain. Surgical treatment was not indicated unless functional improvement was expected even in the more extended position of the PIP joint due to the flexion contracture of the wrist or poor upper extremity function. The inclusion criteria for the study were as follows: (1) presence of a syndrome or other deformity, (2) availability of both pre- and post-operative posteroanterior radiographs of the finger, and (3) adequate information on the flexion contracture and range of motion of the PIP joint. Patients were excluded when (1) the duration of the follow-up was less than two years, and (2) a concomitant Z-plasty of the volar skin was required to obtain a full extension of the PIP joint (Figure 2). Finally, forty-nine fingers (20 on the right hand and 29 on the left hand), representing 24 patients, were included. There were 12 boys and 12 girls. The most commonly affected finger was the little finger (15 cases), followed by 14 cases of the long finger and 10 cases each of the index and ring fingers. Of the 49 fingers, six fingers had the moderate form (30° to 60°) of camptodactyly, and 43 fingers had the severe form (>60°), according to Siegert’s classification [3]. Twelve patients had the procedure performed on multiple fingers, and all fingers with moderate deformities were included. Three fingers had been subjected to previous soft tissue surgery performed by another surgeon. The syndromes associated with the patients and patient demographics are summarized in Table 1.

The mean age at the time of surgery was 8.5 ± 3.7 years (range, 2.3 to 13.4 years), and the mean duration of follow-up was 3.9 ± 1.7 years (range, 2.6 to 11.4 years). The clinical results were evaluated using the degree of flexion contracture and the active arc of motion of the PIP joint. These parameters were measured before the index surgery and at the latest follow-up with the wrist and metacarpophalangeal joints placed in the neutral position. The shortened length of the proximal phalanx was measured using posteroanterior radiographs.

### 2.1. Surgical Technique

**Figure 3:**
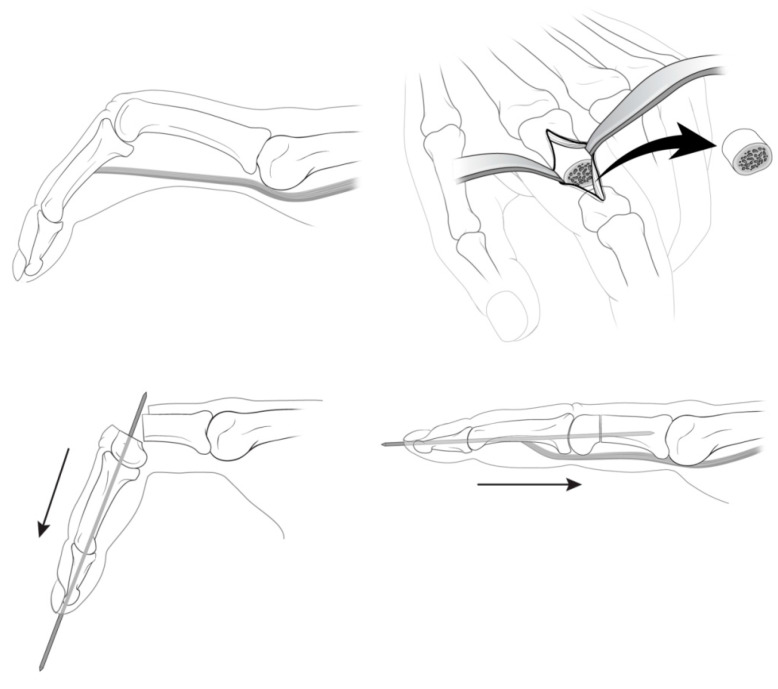
Diagrams of a one-stage extension shortening osteotomy. A preoperative flexion contracture of the proximal phalanx was observed. After a longitudinal incision on the dorsal aspect of the index digits, the extensor digitorum tendon was exposed and split. The periosteum was incised and retracted using Hohmann retractors, both medially and laterally. Two transverse osteotomies between the shaft and the head of the proximal phalanx were performed. The antegrade insertion of a K-wire into the distal fragment was done through the PIP joint. The K-wire was advanced proximally across the osteotomy site to fix while maintaining the reduction.

The surgical procedure is summarized in Figure 3. With the patient in the supine position under general anesthesia and with tourniquet control, the procedure was performed using an image intensifier. A linear or zig-zag skin incision was made on the dorsum of the PIP joint. As the dissection was deepened, the extensor digitorum tendon was exposed and split to expose the neck of the proximal phalanx. The periosteum was then incised at the mid-portion using a blade and retracted using Hohmann retractors both medially and laterally. Two transverse osteotomies were performed at the boundary between the shaft and the head of the proximal phalanx using an oscillating saw. A distal osteotomy was performed perpendicular to the axis of the proximal phalanx’s neck to correct the angulated head. The proximal phalanx was shortened to obtain the full extension of the PIP joint. The length of shortening was determined during the surgery, not preoperatively, by the overlap of the two segments between which of the PIP could be fully extended. Care should be taken not to shorten more than as is necessary for the full extension of the PIP joint. The second osteotomy was performed perpendicular to the proximal phalanx. With the PIP joint fully extended, the antegrade insertion of a Kirschner wire (K-wire) was made into the distal fragment through the PIP joint, exiting the tip of the finger. The K-wire was pulled out toward the distal end of the osteotomy site. While maintaining the reduction state manually or using the K-wire as a joystick, the K-wire was advanced proximally across the osteotomy. An additional K-wire was adjusted to increase the stability of the osteotomy site, as needed, and crossed pins could also be used depending on the surgeon’s preference. Care should be taken not to rotate the osteotomy site. After checking the stability and position of the K-wire under the image intensifier, the incision was closed layer by layer.

Postoperatively, patients were immobilized in the intrinsic plus position with a short arm cast for 6 weeks generally. The cast and K-wire were removed when the proper union of the osteotomy was confirmed, and rehabilitation was used to restore both passive and active movement.

### 2.2. Statistical Analyses

Statistical analyses were performed using SPSS^®^ software, version 25.0 (SPSS, Chicago, IL, USA), with significance defined as a *p* value less than 0.05. Data were assessed for normality using the Shapiro-Wilk’s test. The paired *t*-test and Wilcoxon signed-rank test were used to compare preoperative and postoperative variables.

## 3. Results

**Figure 4:**
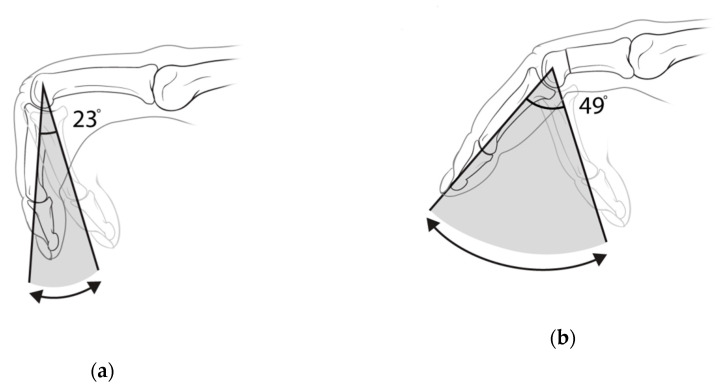
The active arc of motion of the PIP joint, shown (**a**) preoperatively and (**b**) postoperatively.

The mean flexion contracture of the PIP joint improved from 76° preoperatively to 41° postoperatively during passive extension (Table 2). The mean preoperative active arc of motion was 23°, which improved to 49° at the final follow-up (Figure 4). The mean length of the shortening on the proximal phalanx was 4.9 ± 2.3 mm, which averaged 17.8% of the proximal phalanx’s original preoperative length. The mean duration of surgery was 25.8 min, and the PIP joint was fixed using K-wires with an average flexion position of 7.6 ± 14.0°. There were no statistical differences in postoperative outcomes between the little, ring, long, and index fingers.

There were no postoperative complications, such as surgical wound problems, neurologic deficits, malunions, or nonunions. Bone unions were achieved in all cases within six-to-eight weeks after surgery. Six fingers from two patients underwent additional procedures because of the aggravation of the flexion contracture related to a rapid growth spurt; four fingers from one patient were subjected to a second index surgery, and another patient underwent a Z-plasty of the volar skin at postoperative six years.

## 4. Discussion

The pathogenesis of camptodactyly has been defined, based on operative findings, as tightness or shortness of the flexor tendon [2], abnormal insertion of the lumbrical muscle [18], contractures of the surrounding soft tissue, volar skin deficits [12], anomalies in the extensor mechanism, and skeletal changes in the PIP joints [13]. Based on this information, most authors have attempted to correct camptodactyly by releasing the soft tissues. The main topic of previous studies was the identification of the structures that should be corrected to eliminate these anatomical causes. However, it is not easy to surgically release all of the structures involved in the deformity, especially in syndromic camptodactyly, which has a different etiology from idiopathic camptodactyly and affects multiple fingers with more severe contractures [8,11]. The outcomes of soft tissue surgery are dependent on the surgeon’s skill and which structures are released. Further damage to the soft tissues can lead to poor results due to the formation of scar tissue and volar skin adhesions [3,11]. Lengthening or tenotomy of the flexor tendon may cause disturbances in tendon balance and a reduction in grip strength [3]. To avoid these problems, we carried out a one-stage extension shortening osteotomy without concomitant soft tissue release for the treatment of camptodactyly.

Generally, flexion deformities of less than 30–60° do not interfere with daily life and do not necessarily require surgical treatment [3,10,11,19]. We performed surgery for severe or multiple of camptodactyly. In our study, the mean flexion contracture (from 76° to 41°) and the active arc of motion (from 23° to 49°) were both improved significantly. Previous studies reported 10° to 54° improvement in the flexion contracture after soft tissue surgery [3,6,7]. However, two studies that reported better results had a short follow-up period of fewer than 1.4 years. Furthermore, we included only syndrome camptodactyly with moderate or severe deformity, while previous studies were limited to cases of idiopathic camptodactyly involving the little finger alone or only included a small number of cases.

The active arc of motion is as important as the position of the PIP joint for hand function, and the loss of flexion after surgery is a significant complication [3,5]. While some studies demonstrated improvements in the flexion contracture [5,6,13], no intermediate or long-term study documented an increase in the active arc of motion after surgery. Siegert et al. [3] reported that more than 50% of patients complained about a loss of flexion after surgery. Additionally, previous studies reported that, to correct camptodactyly, a release of the FDS was usually performed [3,4,5,6,7,12,13,19,20]. However, the excessive lengthening or tenotomy of the FDS gives rise to a decrease in the active arc of motion and a loss of flexion, even if the procedure was able to provide a better extension of the PIP joint [7]. The choice not to perform concomitant FDS lengthening, thus leaving the FDS intact, could explain the improvement in the arc of motion in this study.

**Figure 5:**
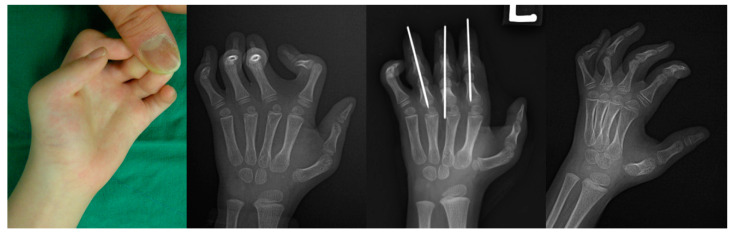
The clinical image and radiographs of an arthrogryposis patient with syndromic camptodactyly show the flexion contractures from second to fifth finger with the left hand’s thumb-in palm deformity. Despite the restricted wrist motion with the lack of wrist crease, the patient did not present the flexion contracture of the wrist but complained of discomfort when grasping objects such as cups. After the correction of camptodactyly via one-stage extension shortening osteotomies, the hand function improved with the proper bony union.

Complicated surgical techniques involving various structures prolong the duration of surgery, which can increase the risk of postoperative infection and surgical wound issues [21]. Furthermore, syndromic camptodactyly frequently involves multiple digits, and a bloodless and straightforward surgical technique with a short operative time would be proper. It is hard to correct flexion deformities involving multiple digits in less than 2 h, which is accepted as the safe limit for tourniquet application [22]. While none of the previous studies described the surgical duration for performing soft tissue release, an osteotomy does not require a long incision, soft tissue reconstruction, or skin grafting, so we can easily expect a shorter surgery. In this study, the mean duration of surgery from incision to closure was only 25.8 min per finger. This technique can thus be performed in a shorter time than soft tissue release and is suitable for surgery on multiple fingers in patients with syndromic camptodactyly (Figure 5).

The benefits of this technique are maximized for relapsed flexion contracture after previous surgeries. The osteotomy technique used in this study does not damage volar structures or soft tissues, so there is no formation of scar tissue or adhesions. The long incision and deep dissection required for soft tissue release inevitably cause some degree of postoperative scar contracture, which can aggravate the progression of the flexion contracture over time. It is not easy to perform a second soft tissue release due to the formation of scar tissue and adhesions in cases of relapsed flexion contracture after previous surgery. However, the revision of volar soft tissue release or an osteotomy can be carried out easily after the procedure. In very severe cases, a combination of an osteotomy and volar soft tissue release can be performed to achieve a satisfactory finger position. In two excluded cases with insufficient correction after an osteotomy due to volar skin deficits, we performed concomitant Z-plasty and obtained a full extension of the PIP joint.

In this study, no patient was able to maintain an immediate postoperative correction until the last follow-up. Even though we achieved a nearly full extension of the PIP joint during surgery in most cases, the flexion contracture tended to progress gradually after surgery. Camptodactyly is a progressive deformity that changes throughout a patient’s growth and development. While camptodactyly can progress after any surgery to correct it, the osteotomy technique has the advantages of a more comfortable revision operation.

The osteotomy technique brings to mind the extension lag due to the loosening of the extensor tendon and cosmetic issues regarding the abnormal lengths of the fingers caused by the shortening of the proximal phalanx. However, the mean length of the shortening is close to the standard deviation of the length of the proximal phalanx of average adults [23]. Based on a previous study of distal finger extension patterns [24], there may be a change in finger length patterns between the index and ring fingers, but other finger length patterns are not typically altered. Additionally, considering the potential for additional flexion contracture after surgery, there is no need to be concerned about cosmetic issues due to a shortened digit length.

There were several limitations to this study. First, our results with the osteotomy technique were not compared to patients with camptodactyly treated only with soft tissue release. However, these soft tissue surgeries cannot be easily compared because the surgical procedures for releasing the involved structures vary widely. Further, also we did not describe the efficacy of the index surgery for idiopathic cases. We have not recommended surgical treatment for idiopathic camptodactyly without discomfort, but this technique may be useful for cosmetic improvements. Second, the posteroanterior radiographs were taken with the PIP joint extended and attached to the detector as much as possible, but the flexion contracture can interfere with the standardized posture for taking radiographs. Even though there can be a measurement bias in the shortened length of the proximal phalanx, the more important finding is the improvements in the arc of motion and the contracture, not merely the length. Third, there is no guideline for the proper age of surgical correction to the best of our knowledge. In another aspect, we could not decide which one is better between early surgery for functional development and late surgery to avoid repeated surgery due to a recurrence. Finally, we did not evaluate the objective measurements of fine and gross motor function and the functional improvements such as grip power or the quality of life of the patients because of the limitation of the retrospective study. However, the function of patients with neuromuscular disease is incomparable to that of healthy people. A further prospective study with age, disease, and severity matched design would be helpful to evaluate the functional improvements after correction of syndromic camptodactyly.

## 5. Conclusions

The use of a one-stage extension shortening osteotomy is straightforward and appealing for the correction of camptodactyly by lengthening all volar structures and simultaneously correcting bony abnormalities. This technique does not damage the volar soft tissues, which can lead to the formation of adhesions and scar tissue. It is suitable for surgery on multiple fingers and can be carried out to treat relapsed camptodactyly after previous surgery. The ultimate goal of surgery for syndromic camptodactyly is to reconstruct functional fingers rather than just a removal of the etiological cause. This technique should be considered a first-line treatment for the correction of syndromic camptodactyly affecting multiple fingers.

## Figures and Tables

**Table 1:** Patient demographics.

Demographic	Value
Age at surgery ^1^ (years)	8.5 ± 3.7
Duration of follow-up ^1^ (years)	3.9 ± 1.7
Sex ^2^	
Male	12 (50%)
Female	12 (50%)
Surgery on multiple fingers ^2^	12 (50%)
Related syndrome ^3^	
Arthrogryposis multiplex congenita	40 (81.6%)
Other syndrome	9 (18.4%)
18 trisomy syndrome	4
Loeys-Dietz syndrome	3
Turner syndrome	1
Unknown origin of developmental delay	1

^1^ The values are given as the mean with the standard deviation. ^2^ The values are given as the number of patients with the percentage in parentheses. ^3^ The values are given as the number of digits with the percentage in parentheses.

**Table 2:** Clinical assessment of the proximal interphalangeal joints prior to surgery and at the final follow-up.

	Preoperative	Final Follow-Up	Difference	*p* Value
Flexion contracture (°)	75.8 ± 19.5	41.1 ± 22.6	−34.6 ± 26.4	<0.001 ^1^
Active flexion (°)	106.4 ± 13.6	96.5 ± 12.0	−9.9 ± 16.6	<0.001 ^1^
Active extension (°)	83.1 ± 18.6	48.0 ± 21.7	−35.1 ± 24.5	<0.001 ^2^
Active arc of motion (°)	23.4 ± 21.7	48.5 ± 21.6	25.2 ± 29.7	<0.001 ^2^

The values are given as the mean with the standard deviation. ^1^ *p* values were derived by the Wilcoxon signed-rank test. ^2^ *p* values were derived by the paired *t* test.
